# The Local Turn: an Introductory Essay Revisiting Leadership, Elite Capture and Good Governance in Indonesian Conservation and Development Programs

**DOI:** 10.1007/s10745-016-9831-z

**Published:** 2016-06-01

**Authors:** Carol Warren, Leontine Visser

**Affiliations:** Asia Research Centre, Murdoch University, Perth, Australia; Wageningen University, Wageningen, The Netherlands

**Keywords:** Leadership, Elite capture, Good governance, Community-based resource management, Indonesia

## Abstract

The local turn in good governance theory and practice responded to critiques of the ineffectiveness of state management and the inequity of privatization alternatives in natural resource management. Confounding expectations of greater effectiveness from decentralised governance, including community-based natural resource management, however, critics argue that expanded opportunities for elite capture have become widely associated with program failures. This overview of theoretical controversies on leadership, patronage and elite capture is part of a themed section in this issue that challenges assumptions across a wide range of current policy literature. It introduces a set of Indonesian case studies that examine practices of local leaders and elites and seek to account in structural terms for appropriations both *by* (‘elite capture’) and *of* (‘captured elites’) these key figures. These studies explore the structural factors and co-governance practices most likely to promote effective participation of the full spectrum of local interests in pursuit of better local natural resource governance.

## Good governance and the local turn

A substantive policy literature has emerged over more than a decade on the role of social capital, community empowerment and local leadership in conservation and development programs (Jentoft *et al.*^1998^; Woolcock 1998; Pretty and Ward 2001; Pretty *et al.*^2003^; Pretty and Smith 2004; Lehtonen 2004; Bebbington *et al.*^2006^; Mosse 2008; Plummer and Fitz Gibbon 2006; Pretty 2006; Dasgupta and Beard 2007; Bodin and Crona 2008; Woolcock 2010). This literature is part of a wider discourse on ‘good governance’, which regards transparency, accountability and social inclusion as basic building blocks to equitable development and sustainable resource management outcomes (World Bank 2004; Batterbury and Fernando 2006). With a strong focus on enhancing the role of civil society through expanded public participation and empowerment, decentralization of governance became a global aspect of the new strategy (World Bank 2002).

The turn to the local in good governance theory and practice moved more recently from mainstream debates in development policy circles into conservation literature^1^. There it met up with a longer standing body of ‘commons’ scholarship reacting against dominant readings of Hardin’s (1968) tragedy of the commons allegory that had placed concerted policy emphasis on the supposed advantages of state and private resource management (Ostrom 1990; Berkes and Folke 1998; Agrawal and Ribot 1999). This earlier shift of focus away from the mainstream predisposition toward centralised resource management to community institutions in common property scholarship and non-government organization circles responded to critiques of the ineffectiveness of state management of resources and the inequity of privatization alternatives (Ostrom *et al.*^2002^; Warren and McCarthy 2009; Cleaver 2012). These debates were particularly relevant to the management of apparently ‘open access’ forest and marine resources of the archipelagic and (once) heavily forested nation-state of Indonesia (Vandergeest and Peluso 1995; Jentoft *et al.*^1998^; Visser 2004; Lowe 2006; Cribb and Ford 2009).

Neither public accountability nor efficient use of natural resources was evident in the effective enclosure that resulted from either private or state resource monopolies. Both state and private appropriation of environmental goods had the effect of excluding resource dependent communities that claimed legitimate rights to natural resources on the basis of precedence and customary stewardship. Arguing that local communities or user groups have both the local knowledge and ongoing interest in the sustainability of these resources by virtue of direct dependence, the community-based turn in conservation and development interventions became an increasingly strong trend in policy circles over recent decades (Agrawal and Gibson 2001; Brosius *et al.*^2005^; Grafton 2005; Berkes 2007).

In Indonesia the local turn coincided with reformist moves toward political democratization and decentralization and the weakening of the state’s role in both conservation and development policy spheres after the fall of the Suharto regime in 1998 (Aspinall and Fealy 2003; Patlis 2008; Hadiz 2010). Consequently, social and environmental NGOs found themselves uncomfortable bedfellows with neoliberal proponents of decentralized governance from the World Bank and other global conservation and development agencies in community interventions ostensibly aimed at accountability and community empowerment (Harriss 2001; Hadiz 2004; Mansuri and Rao 2013). Pursuant to the local turn in conservation circles, social research and policy debate has focused on how to engage community-wide participation and ensure equitable sharing of the costs and benefits of natural resource management,^2^ particularly among the most marginalized social groups – women, minorities, the poor – in order to protect both environmental values and human rights, which became ever more closely interdependent in global discourse.

The results of these community-based interventions have been equivocal, however. Community-based natural resource management (CBNRM), began to confront problems arising from an idealized model of ‘community’ structures and a lack of appreciation of the political and economic inequalities that had obscured the differential effects of programs whose benefits and burdens were not fairly distributed and often led to perverse outcomes (McCay 2001; Veron *et al.*^2006^; Bené *et al.*^2009^). The result is that the pendulum swing in good governance theory and policy from centralized orientations of the 1970s and 1980s, to international enthusiasm for decentralization in the following decades, now seems to be groping toward some middle ground involving nested governance and multi-stakeholder co-management approaches (Ostrom 2010; Marschke 2012: 16–22).

## Neo-Liberal Good Governance Discourse and the Community-Based Agenda

Critics point to significant gaps, ambiguities and contradictions in the theoretical frameworks and case-study evidence surrounding community-based conservation and development interventions that indicate serious disjunctions between theory and practice (Harriss 1997; Fine 2001; Harriss 2001; Bebbington *et al.*^2004^; Gray 2005; Li 2007; Mosse 2008; Mansuri and Rao 2013; Saunders 2014). Key areas of continuing debate are the focus of unresolved dilemmas for conservation and development programs: First of all, there are fundamental tensions between the goals of inclusive socio-economic development and conservation strategies, because most natural resource dependent growth is ultimately implicated in resource depletion. Regulated restraint aimed at conservation typically falls on those most directly dependent on the resource and least able to resist effective ‘enclosure’ by government, the private sector, or international conservation agencies’ management strategies. At the same time, resource depletion also hurts the poorest users most, since those with substantial capital can more readily shift their enterprise elsewhere.

Secondly, persistent failures to achieve either conservation or equitable livelihood objectives through so many of these community participation initiatives led critics to question assumptions that institutional design can provide straightforward solutions to natural resource management issues. Not least, critical attention from political economy and political ecology research reminds us that local power structures are often highly asymmetrical. As a consequence elite domination has become the focal point of much debate concerning limitations of community-based conservation and development programs (Hadiz 2004; Platteau 2004; Bebbington 2007; Dasgupta and Beard 2007; Bené *et al.*^2009^; Lund and Saito-Jensen 2013).

Research suggests a disturbing but predictable association between pre-existing structural inequalities and the ineffectiveness of socio-economic development and environmental protection interventions. These failures are widely attributed to capture^3^ of institutions and resources by elite interests (Bardhan 2002; Platteau 2004). Platteau writes, local elites “tend to appropriate a larger share of the transfers in communities that are highly unequal to begin with” (2004:230). More positively, some authors assert a strong relationship between levels of broad political participation and the potential for constraint on elite capture at all scales of governance (Bardhan 2002:194; Mansuri and Rao 2013:10), an argument that is pursued in the collection of case studies that follows.

This essay introduces case studies on the role of leadership and local elites in resource governance in Indonesia, and their implications for wider debates on leadership, elite capture and good governance. Several of our cases suggest structural conditions in which ‘captured elites’ may be an appropriate descriptor for situations in which social relationships and expectations drive leaders to instigate or support collective actions well beyond self-interested considerations. Fritzen (2007), concluding his analysis of 250 community-driven development projects in Indonesia, finds that despite the pervasiveness of elite control of decision-making boards, the degree of elite involvement bore no direct relationship to capture of project benefits. Rather, he found ‘… the degree of democratic selection of community boards emerges … as a consistent, significant predictor of pro-poor, pro-accountability dispositions and competent boards’ (Fritzen 2007:1370).

Nonetheless, considerable obstacles stand in the way of genuine democratic practice at all scales of governance. While opportunities disproportionately avail to local elites by virtue of political connections to external traders and government agencies, it is also the case that ordinary villagers sometimes benefit from collusive behavior. Reluctance of local leaders to strictly enforce internal community sanctions for violation of resource management rules or to take collective action to resolve generally understood local environmental crises is a notable feature of governance arrangements where personal and community relationships might be jeopardized by strict law enforcement, as shown in several of the studies of leadership and elite capture in this themed section.^4^

Finally, associated with the question of entrenched hierarchy and inequality in the local domain has been evidence of the exclusions or at the very least intractable constraints on the scope for agency among marginalized groups who are likely to be bypassed in economic development interventions and disproportionately affected by new regulatory conservation regimes (Adhikari and Goldey 2010). In this regard, the extent of legitimate and representative leadership and the search for mechanisms to respond to elite capture in their absence become central to these ongoing debates (Arnall *et al.*^2013^:309; Laerhoven and Ostrom 2007:11; Lund and Saito-Jensen 2013).

## Leadership and Elite Capture: Debates

The articles that follow in this volume focus on the extent to which local leaders and elites use their roles to ensure that natural resources serve broad, long-term common good outcomes. The concept of leadership in the literature generally refers to people who may be elected or appointed to formal positions in governance structures, whether traditional forms of governance (customary *adat* in the Indonesian context) or state structures of public administration from local to national level. References to local leaders here generally refer to those who hold an acknowledged position within traditional or state governance structures at hamlet and village levels (Uphoff 1986:11; Arnall *et al.*^2013^) or who hold positions of respect and influence based on social or economic roles. Concepts of leaders and elites are regularly used interchangeably in the literature, on the assumption that local leaders are typically if not always drawn from established and/or advantaged economic and social status groups (Dasgupta and Beard 2007:238). Instead of automatically conflating leadership and elite status, or taking rent-seeking interests of the local elite for granted, the papers in this issue look at the articulation between horizontal and vertical power relations, and consider internal alliances and frictions among local elites, as well as engagements with external actors and institutions, particularly in relation to natural resource management.

The collection of case studies presented in this issue also connects with debates in the development and conservation literature on ‘social capital’ and the structural conditions needed to achieve sustainability and equity ends. Contributors to these debates tend to diverge along lines we might describe as pragmatic policy-oriented versus critical theory approaches: the one strand follows Putnam *et al.*’s (1993) interest in the capacities of social groups to use horizontal associations and normative values to achieve institutional bases for collective action and common good outcomes. The second, using a particular reading of Bourdieu’s (Bourdieu 1990[1980]; 1986) approach to social capital, focuses on the more instrumental use of social and symbolic resources by individual actors and social groups to achieve advantage (Harriss 1997, 2001; Fine 2001).

Application of the Putnam approach feeds into research that is focused on community capacity for common good collective action, and tends to treat leadership in its functional role, predicated on local social norms and reciprocal relations. Allied approaches include the moral economy literature investigating the bases of social solidarities and subaltern strategies that revolve around social security, reputation and mutual aid (Scott 1976; Cleaver 2012), as well as policy literature that assumes the functional necessity of engaging effective representative leadership through institutional design (Ostrom 1990; Fritzen 2007). Nonetheless, as Uphoff (1986:10) argues, what is called a ‘community’ may or may not provide a substantial social basis for collective action. In the Indonesian context explored in this themed issue, the *adat* community may include the whole customary village (as in the Bali case) or may be differentiated into the core and non-core families of the village (as in the Moluccas case). ‘Community’ as the basis for collective action need not be a geographically defined entity, but may refer to the more dispersed context of a patron’s network or clientele (as in the Kalimantan case).

Bourdieu’s (Bourdieu 1990[1980]; Bourdieu 1986) approach on the other hand concerns the reproduction of hierarchy and advantage through deployment of social and symbolic forms of capital that privilege strategically positioned and culturally sanctioned status and wealth groups. The transformation of symbolic capital into economic capital, and vice versa, where structure and agency are mutually dependent, is also key to our understanding of the role of local leadership and patronage in present-day Indonesian societies. Bourdieu’s approach to elite domination has become the foundation of a body of literature focusing on the reproduction of power hierarchies and their effects on governance. It underpins elite capture assumptions and seeks to explain how ineffective and inequitable policy outcomes persistently result from asymmetrical structural characteristics of communities and social groups (most explicitly evidenced in the Sumatran case). However, as Bebbington (2007:160) argues, this focus on vertical power relations obscures the existence of other ‘disinterested’ and cooperative forms of local leadership and social action based on horizontal solidarities.

## Focus on Indonesia

**Fig. 1:**
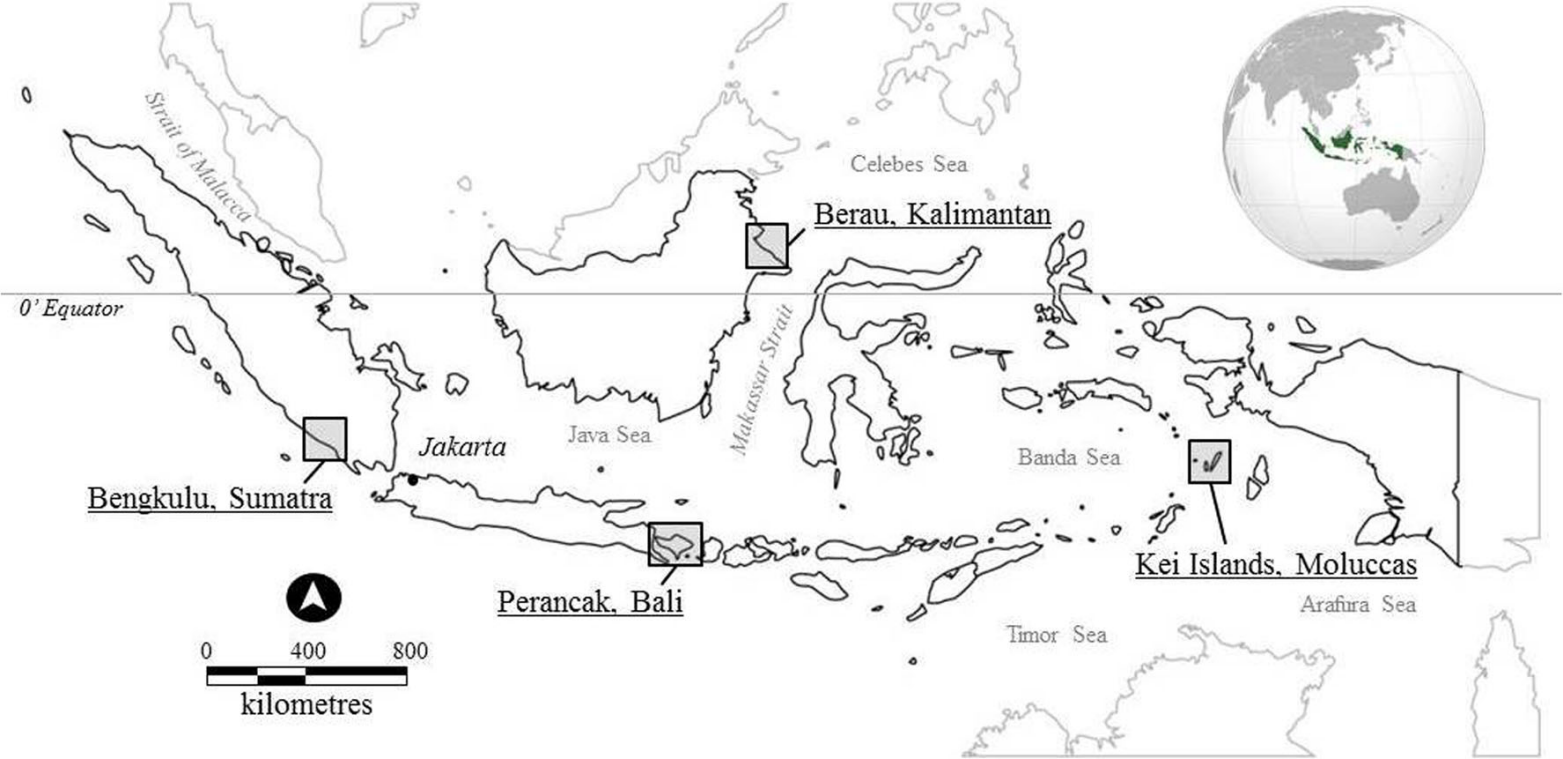
Map of Indonesia: Case study locations

The case study articles that follow analyze the roles and positions of local leaders and/or elites with regard to critical local resource governance issues in Indonesia (see Fig. 1 for case study locations). Indonesia presents a rich ground for comparative case studies. The Indonesian cases we explore represent a diverse set of cultural and environmental contexts within the framework of a single rapidly developing and democratizing nation-state that has committed to a dramatic decentralization of governance as part of the reform process since the fall of the Suharto regime in 1998 (Aspinall and Fealy 2003; Hadiz 2010; Satria *et al.*^2006^; Patlis 2008). This concentration on single-country case studies enables us to look in depth at questions of leadership and elite control within the same formal state governmental framework, in local contexts that at the same time present different types of ‘community’, with different ethnic compositions, different local histories and different resource bases. This formal legal baseline enabled us to address Agrawal’s (2002) call to explore the gap in understanding how the complexities of context explain differential outcomes.^5^

In tandem with the reconfiguration of governance through decentralization in the post-Suharto reform era, villages across Indonesia have been subjects of a concerted program of community-based empowerment interventions prompted by the World Bank’s Social Capital Initiative (World Bank 2002). This community-driven approach to local development has been taken up by the Yudhoyono government and implemented across the country under the label of the National Community Empowerment Program (PNPM) (Bebbington *et al.*^2006^; McCarthy *et al.*^2014^). PNPM provided a pan-Indonesian intervention designed to broaden the role of civil society in order to maximize community involvement and minimize elite capture. Among other objectives, the research reported in this collection of case studies investigated the extent to which this and analogous community-based interventions in local conservation as well as development programs succeeded in enhancing public engagement in local governance, and whether participatory reforms translated into better resource management and fairer distribution of benefits.

The twin processes of decentralization and democratization of governance in Indonesia since the dramatic fall of the Suharto regime in 1998 – that was partly catalyzed by emerging human rights and environmental movements – offered opportunities to put discourses of community participation and empowerment into practice (Eldridge 1995; Hirsch and Warren 1998; Peluso *et al.*^2008^; Warren and McCarthy 2009). There has been an implicit assumption accompanying the good governance focus in international policy circles that political decentralization would bring management closer to those who directly depend on natural resources, stimulating conditions for improving the position of previously marginalized groups as well as degraded environments (World Bank 2002; Bardhan 2002; Batterbury and Fernando 2006; Ribot *et al.*^2006^). Scholars and policy makers assumed that at local scales it would be easier to build on local knowledge and synergies and to tighten the links between incentives and accountabilities (Resosudarmo 2004; Patlis 2008). Evaluations of these local participation and empowerment programs have been mixed, however. Confounding expectations of efficiency and effectiveness from democratized and decentralized governance, including community-based natural resource management, evidence of expanded opportunities for elite control and capture have become widely associated with decentralization policies (Aspinall and Fealy 2003; Blaikie 2005; Brosius *et al.*^2005^; Wardell and Lund 2006; Hadiz 2010).

## Leadership in Community-Based Conservation and Development Programs

With respect to the environment, the need to internalize access rights and responsibilities for the protection of natural resources in order to avoid the proverbial tragedy of the commons was a prominent trope underpinning decentralization and community-based management policies and practices (Warren and McCarthy 2009; Adhikari *et al.*^2014^). Some evidence of better targeted service delivery as a result of decentralized and participatory governance experiments has been reported among reviews of community development programs (Bardhan 2002; Bebbington *et al.*^2006^), although not the thoroughgoing institutional transformations or empowerment that had been predicted earlier (Batterbury and Fernando 2006:1853; Bené *et al.*^2009^). The picture is yet more qualified for community-based environmental governance interventions. Because conservation programs typically require restricted or deferred benefit, different dynamics may be invoked and different leadership qualities required. Indeed, trade-offs between social and environmental protection are difficult to avoid (Wardell and Lund 2006).

Non-government organizations often act as brokers in negotiating these trade-offs between state, corporations and local communities or user groups – toward local resource access (the primary focus of grassroots and human rights NGOs) or environmental protection goals (typically the concern of global conservation agencies). They tend to seek engagement with different types of leadership and at different scales, depending upon their donor support base (see, for example, Steenbergen 2013; Kusumawati, 2014). Environmental protection goals have been rather heavy-handedly pursued by the large international conservation agencies whose impacts can be equal to those of governments in some regions (Batterbury and Fernando 2006:1856; Afiff and Lowe 2008).

Local elites may be less able to avail themselves of rent-seeking opportunities through conservation interventions (typically aimed at restricting resource access) than is the case with externally funded programs aimed at community development. Nonetheless, established local figures may seek to enhance their authority as brokers for NGO environmental projects in an effort to appropriate institutional resources and build reputation and networks. For their part, external partners (NGOs, state agencies and private corporations) find it difficult to commit to time-consuming local participation processes and may be inclined to engage whatever legitimacy local customary or official administrative leaders possess in a mutually bolstering process.

## Case Studies of Leadership and Participation in Local Resource Governance

The case studies presented in this volume are based on grounded, long-term field research across a range of Indonesian societies and resource bases in Sumatra, Kalimantan, the Moluccas and Bali (see locations Fig. 1).^6^ They address questions of leadership and participation in local resource governance and in empowerment programs introduced by central government and non-government organizations. They give attention to the inclusions and exclusions of particular social groups and roles, as well as the conditions accounting for effective or perverse outcomes in resource conservation and development programs. The studies explore the characteristics of local customary leaders, political and economic elites as well as investigating how they deploy available forms of local power, authority, influence and representation.

The case studies seek to account in structural terms for appropriations both *by* (elite capture) and *of* (captured elites) these key figures, and to identify the kinds of leadership and co-governance practices most likely to accommodate the sometimes conflicting implications of conservation and development programs, and to promote effective participation of the full spectrum of local interests and identities. Their arguments resonate with other recent studies that have pointed to the need to harness elite skills and resources and explore what accounts for ‘benevolent’ engagement as well as ‘pernicious’ capture of project benefits and decision-making mechanisms (Fritzen 2007; James 2011; Knudsen 2013).

The account by Lucas of corruption in two Sumatran case study villages describes the classic condition of elite capture of development project funds through systematic rent-seeking behavior that features so prominently in the aid and development literature. The cuts, kickbacks and other illegal payments from project interventions blatantly demanded by local officials replicate a model of rent-seeking that dominated central government cronyism in Indonesia under the centralized and authoritarian Suharto regime. Indeed the reform process in Indonesia is widely regarded as having been hijacked by the decentralization of corruption through the ‘money politics’ that have dominated the reform era since 1998 (Aspinall and Fealy 2003; Hadiz 2010). The two Sumatran cases described by Lucas involve examples of nepotism, misappropriation and collusion by village officials with government superiors and local clients. Nonetheless, in one of his two cases, factional divisions and competition among the village elite counteracted corrupt tendencies and managed to serve public interest reasonably well in the instance of a water supply project. Local officials also collaborated with an Indonesian conservation NGO in reinstating and adapting customary resource protection regulations, now recognized alongside the official village administrative framework.

The Kusumawati–Visser study of a turtle conservation program in coastal Kalimantan is focussed on patron-client networks rather than on ‘village communities’. It looks critically at patronage in its broader social context, as opposed to the narrowly political and economic frameworks that almost by definition treat the asymmetrical dimensions of these relationships as impediments to conservation and development program success. The authors argue that the narrow focus of international donors on ‘elite capture’ glosses over the internal diversity and political–economic differentiation of interests among members of local elites. It also risks losing the potential for ‘capturing the elite’ and recruiting the social and historical authority, environmental knowledge, organizational skills and redistributive capacities of economic patrons, thus precluding the possibility of partnering with their resource-dependent clients. Kusumawati and Visser take a more constructive approach to dealing with local hierarchies in the Kalimantan case. They ask how the positive aspects of patronage relationships, so pervasive in artisanal fisheries in terms of credit, technology and social security provision (Gunawan and Visser 2012), might be accommodated in conservation and management interventions. As Ferrol-Schulte *et al.* (2014:63) argue, “Although patrons have been identified … to be drivers of resource exploitation, they are also potential agents in identifying and activating sustainable solutions to environmental decline and improving fishing household resilience.” The active engagement of trusted patrons might also bring valuable local knowledge and legitimacy to the interventions of outside conservation agents, who typically lack the legal status, credibility and grounded relationships that long-standing local exchange networks could bring to regulatory efforts.

In the Moluccan community studied by Steenbergen in this issue, customary (*adat*) leaders wielded considerable authority, boosted by their role in negotiating NGO– facilitated conservation agreements. It is notable that the community’s customary leadership independently recognized the need for coastal resource protection measures following declines in yield of important marine resources, which ultimately prompted local overtures for partnership with a small Indonesia-based NGO that has a strong commitment to local management. The NGO depended heavily on customary leadership and processes for implementation of new conservation programs. Advantages accrued to the dominant local *adat* group as a result of external agency collaboration that facilitated formal acknowledgement of customary institutions, reinforcing the leadership position of this group. Marginalization of ethnic minorities was a feature of local decision-making here as in the Sumatran cases described by Lucas, although the extent and implications remain open to interpretation.

The Bali case presented by Warren focuses on two dimensions of leadership arising from local horizontal and vertical ties that bind different types of leadership to the wider community in different ways. Political and economic dependencies, as well as local solidarities based on customary community membership, kinship, ethnic and religious identity, bear on an incident of local decision-making failure that did not initially involve external intervention. Indeed, state law enforcement was uncharacteristically regarded as one of the missing links required for resolving several of the major local resource management issues in this coastal community. In her study of the failure of local leaders to gain consensus for restricting seaweed harvesting for the sake of coastal conservation, customary hamlet and fishing cooperative leaders – who are typically regarded as first among equals in contrast to elites within the formal government hierarchy – were unable or unwilling to deploy the various forms of authority associated with their leadership positions to deal with the classic tragedy of the commons situation that unfolded in this Balinese village. The local leaders and political elites of Warren’s study presented themselves as captured by the very bonds that are theoretically assumed to catalyse collective action. Instead they found themselves paralysed, despite widely shared views on the urgent need for environmental rule-making. They were unable to deal with the damaging consequences of the seaweed harvest, until a different conjuncture with state policies produced a changed climate for action.

## Time and Context: Historical and Cultural Interdependencies

The Kusumawati-Visser and Warren studies share Wong’s (2010) concern with the importance of understanding the multiple character of relationships (cultural, social, economic, political) between leaders, elites and non-elites, especially at local level where they are socially embedded by history and proximity. At the same time, both studies find that the character of leaders’ relationships with external agencies and government authorities were crucial contextual aspects of governance that had to be engaged if effective synergies and enduring outcomes were to result. The Bali case demonstrates, on the one hand, that activating horizontal relationships through democratic institutions and associational ties will not automatically assure positive environmental outcomes, at least in the short term. In that case, avoiding internal conflict in an economically depressed context made environmental action for the long term common good difficult for local leaders. Conversely, the Kalimantan case shows that it should not be assumed that asymmetric patronage relations will by definition obstruct progressive social and conservation improvements, and indeed could facilitate them (such as through controlled exploitation of turtle eggs) by keeping political–economic relations transparent, a policy approach that was beyond the scope of the international NGO’s agenda.

In this regard we concur with Arnall *et al.* (2013), whose research on community-driven development programs in Mozambique leads them to conclude that practitioners must take account of the internal diversity and complexity of what is often glossed together as ‘the’ local elite, their contributions, as well as their capacity to derail and divert remedial efforts. In particular, they point to the informal checks and balances that may be rooted in local norms and practices. This point refers us to the implications of the ‘linking’ (vertical) type of social capital which gave local elites in all our cases strategic access to external information and resources that could be activated for the benefit of community and user groups and/or manipulated to their personal advantage in different circumstances. It is only by way of detailed and long-term ethnographic study of local leadership that such checks and balances can be unearthed, or their absence revealed, as shown in the Sumatran cases.

Warren’s work on the horizontal pressures that can be brought to bear by ordinary villagers on local leadership, and Steenbergen’s study of dispersed *adat* leadership are also consistent with Arnall *et al.*’s (2013) stress on the formal and informal constraints that non-elites place on leadership through the ballot box and normative expectations of balanced and generalized reciprocity in certain social contexts. Knudsen (2013) and Bankoff (2015) argue similarly for the importance of recognizing a distinctive personalized type of informal, neighborhood-level leadership that is horizontal, grounded in local values, notions of community service and collective responsibility, one that is not adequately encompassed by patron–client models. Lucas’ case study of systematic corruption in his Sumatran cases, on the other hand, leads us to question assertions by Arnall *et al.* (2013:312) that locally based ‘check and balance’ mechanisms are likely to be more efficient than externally imposed ones. Both the Lucas and Steenbergen cases show that intervening NGO programs can contribute toward expanding the framework of governance toward greater inclusion of women and to a lesser extent ethnic minorities, although Steenbergen shows that NGOs tend to be averse to the risks of becoming involved in internal conflicts because of threat to their donor base. The findings of both the Moluccan and the Kalimantan cases indicate that the interplay between culturally and historically framed horizontal and vertical relationships greatly impact upon the effectiveness of conservation interventions.

It is clear from these case studies that time and context dynamics – including those prompted by external interventions – may shift processes and outcomes substantially. In their studies of elite capture and participatory initiatives in Tanzania and India, Lund and Saito-Jensen (2013:104) found that “initial elite capture of the participatory initiatives was circumvented over time through various forms of resistance orchestrated by initially disadvantaged groups.” They argue that studies of elite capture should be based on in-depth and longitudinal empirical investigations that carefully characterize forms and outcomes of elite capture and consider both the changing dynamics of social settings and the perceptions held by the people under study.

These studies pursue with ethnographic depth the changing constellations of relationships between local leaders/patrons/elites and those whose interests they claim to represent in contexts of resource decline and varying degrees of external government and non-government organization intervention. They also fill an important gap by including cases of the still rather understudied issue of local governance in rapidly declining marine resource contexts.

## Concluding Considerations

The studies of elite capture and leadership in this Themed Section expose the changing dynamics of decentralized local resource management in diverse Indonesian contexts. Nested, transparent and accountable^7^ governance based on democratic and inclusive decision-making at all scales appears to be a basic pre-requisite that is necessary if not sufficient for the transformation of exploitative scenarios linking human and natural resources. The kinds of leadership that emerge from the case studies presented here characterize the leadership-elite capture spectrum, from progressive examples of responsive leadership, at the one end, to outright corruption at the other extreme, and in between by embedded patronage networks and captured elites whose commitments and capacities to lead may be directed or compromised in the interplay between horizontal and vertical pressures and between personal and collective, short- and long-term interests. Not uncommonly, the same actors display several types of agency over time and in different contexts. Collective identities and personal agency often prove ambivalent forces in the commons dramas that emerge from this interplay and that are particularly acute in marine and forest resource dependent communities, such as those described in these case studies.

While the systematic misappropriation of development project funds in the Sumatran case reflected a particularly predatory form of elite capture, the customary leaders who were the key figures in the Moluccan community apparently devoted their considerable cultural and social capital to both conservation and community development goals. Here, local leaders’ traditional customary standing as leaders of patrilineal kinship groups was consolidated by their engagement with an environmental NGO. At the same time, this case study describes a form of leadership that was also apparently aimed at preserving and expanding the local ‘commonweal’ (Warren and McCarthy 2009). Collaboration between customary elders and young ‘institutional entrepreneurs’ (Cleaver 2012) effectively engineered external engagements and internal distributions.

The Moluccan case also supports the argument of the Kalimantan study that engaging local leaders and elites as suspect yet potentially valuable brokers for project interventions may help control their practices if effectively ‘captured’ by NGO partners. The capacities and commitments of local leaders and ordinary villagers may be drawn upon (or compromised) by these external partnerships. Fritzen (2007) stresses the importance of democratic selection and serious capacity building in accounting for benevolent engagement of leaderships as opposed to pernicious elite capture. Imperfect as the local interventions intended to extend democratized governance may be, as Arnall *et al*. argue, NGOs “need to recognize and make the most of what accountability mechanisms already exist” (Arnall *et al.*^2013^:328). Our studies give depth and detail to the complex, nuanced and often ambivalent relationships out of which new sensibilities, practices and rules may become established and contribute, through forms of what Cleaver (2012) calls ‘institutional bricolage’, toward more sustainable development.

As decentralized governance in the new millennium brings more power to local leaders there is a critical need to carry out such in-depth, long-term studies of changing power constellations from local to global spheres, and to work toward the capture of elites by strengthening both horizontal and vertical institutional checks and balances. In this regard, an important legislative change in Indonesia’s decentralized local governance regime undoubtedly had regressive effects on the accountability objectives of the original legal reforms. Local councils at village level (BPD), which had initially been fully elected and independent of the executive, were converted to consultative bodies without independent powers. These were once again subordinated to the village head in the 2004 revisions of Indonesia’s 1999 decentralization laws, severely impairing their check and balance role. Further revisions through the new Village Law of 2014 may yet re-establish a balance between executive and legislative arms in local government, however.

Not to be forgotten then, in this consideration of the relation between leaders and followers, vertical and horizontal forces, in local decision-making on resource management, are the national and global forces that articulate with the local. These have become everyday sites of engagement where elites and other local leaders play critical roles in brokering accommodation or resistance to often contending economic development and conservation interests. There is a need to institutionalize more effective internal participatory community monitoring mechanisms to increase transparency and accountability of officials at the village level as well as external authorities at the sub-district and district levels. This will become all the more important as the community development and empowerment funding previously processed through the PNPM program has now been incorporated in modified form into the Village Law of 2014, substantially increasing the power and resources of village level structures.

Undoubtedly, there has been some convergence between reform-minded activist and neo-liberal agendas in rescaling power and resource allocation to local institutions (Hutchison *et al.*^2014^). Established power relations and the mechanisms through which the local domain has been incorporated into asymmetric globalizing political and economic systems undoubtedly pose formidable, but also contestable, structural impediments to achieving inclusive and sustainable social and environmental protection goals. We have attempted in this set of case studies to address the internal dynamics driving leadership in the local governance of resources in the context of profoundly important articulations with national and global forces, government and non-government institutions.

## Footnotes

1 Batterbury and Fernando (2006:1853) note the relative lack of consideration of natural resource issues in the ‘good governance’ literature, except as extensions of development studies findings.

2 Compliance cannot be assured without general perceptions of fairness in process and outcomes, and ultimately trust in the legitimacy and endurance of governance arrangements. Effective inclusion of stakeholders in decision-making processes is argued to be necessary to assure ownership of rules that attempt to match resource access and stewardship (Grafton 2005; Fritzen 2007).

3 Following Dasgupta and Beard (2007), we distinguish between ‘elite control’ over resources and project interventions that provide access to benefits for non-elites, and ‘elite capture’ of project benefits, which refers to ‘the process by which [local elites] dominate and corrupt community-level planning and governance’ (Dasgupta and Beard 2007:230). See Batterbury and Fernando (2006:1853ff) for more detailed coverage of generally accepted definitions of elite capture in the development and environment literature. The concept extends to informal as well as formal spheres of influence and mechanisms of appropriation. See also Lund and Saito-Jensen (2013).

4 See also Steenbergen (2013) and Gunawan and Visser (2012) where village fishing sanctions that apply in principle to both community members and outsiders are in practice only seriously imposed on non-related outsiders.

5 Similar concerns to address the complexities of context are expressed by a number of analysts of community-based resource management and development issues (Bebbington et al. 2006:1963; Fritzen 2007:1373; Laerhoven and Ostrom 2007:11; Lund and Saito-Jensen 2013:104).

6 This research formed part of a wider comparative project on ‘Social Capital, Natural Resources and Local Governance in Indonesia’, funded by the Australian Research Council (DP0880961). The project involved among others, Lucas, Steenbergen, Visser and Warren, whose ethnographic field research forms the basis of the case studies presented in this volume.

7 Veron et al. (2006:1922), based on empirical studies of the Employment Assurance Scheme in rural West Bengal, argue that two-way accountabilities are critical. “When these vertical accountabilities are weak, horizontal accountability structures between local civil society and officials can mutate into networks of corruption in which ‘community’ actors become accomplices or primary agents.”
